# Iran’s research prioritization: Are we meeting the goals? A study based on clinical trial registry data

**DOI:** 10.1371/journal.pone.0301414

**Published:** 2024-04-05

**Authors:** Farshid Fakhri, Mohammad Mohammadi, Sana Eybpoosh, Sharareh Ahmadi, Masoud Solaymani-Dodaran

**Affiliations:** 1 Department of Epidemiology and Biostatistics, School of Public Health, Tehran University of Medical Sciences, Tehran, Iran; 2 Department of Cell and Molecular Biology, School of Advanced Sciences, Medical Branch, Islamic Azad University, Tehran, Iran; 3 Department of Epidemiology and Biostatistics, Research Centre for Emerging and Reemerging Infectious Diseases, Pasteur Institute of Iran, Tehran, Iran; 4 Student Research Committee, Nursing and Midwifery School, Kermanshah University of Medical Sciences, Kermanshah, Iran; 5 Minimally Invasive Surgery Research Center, Hazrat-e-Rasool Hospital, Iran University of Medical Sciences, Tehran, Iran; Lahore University of Biological and Applied Sciences, PAKISTAN

## Abstract

The prioritization of research topics in the health domain is a critical step toward channelling efforts and resources into areas that have received less attention. The objective of this study is to evaluate the implementation of research priorities determined at the national level within Iran for the period spanning five years between 2009 and 2013. We extracted the required data from the Iranian Registry of Clinical Trials (IRCT) website. Then we conducted a matching process between the titles of trials registered in the IRCT until December 3rd, 2013, and the list of national health research priorities in the domains of communicable and non-communicable diseases. The latter was compiled and regulated by the Research and Technology Deputy of the Ministry of Health since 2008. Out of the total 5,049 clinical trials registered in IRCT, 92.3% were carried out within the domain of non-communicable diseases, while 6.1% pertained to the field of communicable diseases and the remaining 1.3% in other fields. 56.4% of the clinical trials conducted in the field of communicable diseases and 32.8% of those conducted in the field of non-communicable diseases were consistent with the research priorities determined in these two fields. During the five-year period of the prioritization goal, there was no significant improvement in adherence to the list of priorities compared to the previous five-year period. Furthermore, certain priorities were neglected within both areas during these periods. It is possible to evaluate the effectiveness of research prioritization using the data obtained from the registration centers of clinical trials. Our study has revealed that the list of priorities has not garnered adequate attention from the research community within the country. Hence, remedial measures are imperative to ensure the priorities are given more attention after publication.

## Introduction

Prioritization of health research through the prevention of resource and effort wastage, along with the allocation of attention towards under-researched domains, holds the potential to mitigate health inequalities [1–3]. Consequently, prioritization has been deemed as significant as the research activity [4,5]. Which is particularly important in the developing countries.

In Iran, the latest national-level prioritization was carried out in 2008 by the Department of Research and Technology of the Ministry of Health, in collaboration with 40 universities of medical sciences. The final list was published in 2010 for a five-year period from 2009 to 2013, consisting of nine different areas including communicable diseases, non-communicable diseases, health system research (HSR), basic sciences, traditional medicine & herbal drugs, environmental health, nutrition, dentistry, and pharmaceuticals and industry [6].

However, prioritization alone does not guarantee the achievement of its objectives, and It is imperative to evaluate the extent of implementation of priorities after their publication (7,8). Through such evaluations, neglected areas can be identified and considered in future prioritization processes, which can subsequently enhance the legitimacy of these priorities. By doing so, policymakers, researchers, and investors are more likely to accept and endorse these priorities [2,7,8].

It can be possible to evaluate the degree of application of priorities by matching studies conducted or costs incurred with predetermined priorities within a given context [7,9]. To identify the neglected research areas, the studies conducted or research expenses on health research are often compared with the burden of diseases [2,8,10,11]. Additionally, since clinical trials represent a substantial portion of healthcare R&D in public and private sectors, some studies employ clinical trials as a criterion for health research activities. Information on these trials was extracted from registration centers for clinical trials [8,10].

Clinical trials should be registered in registration centers in such a way that their information is available to the public, which can have various benefits and applications for different stakeholders, including patients, physicians, researchers, financial sponsors of clinical trials, and other groups in society [12–19]. Clinical trial registration centers are a source of recorded information on thousands of conducted and on-going clinical trials, providing a unique tool for studying clinical trials [8,10,18,20,21].

The Iranian Registry of Clinical Trials (IRCT) was established in 2008 as the only Farsi-English language member of the International Clinical Trials Registry Platform (ICTRP). Since its inception, a significant portion of the clinical trials conducted in Iran have been registered at this center [22]. Although some clinical trials from past years have been registered, which we also used in the present study.

This study aimed to investigate the compliance of clinical trials registered in IRCT with research priorities set in two areas of communicable and non-communicable diseases from 2009 to 2013. In addition to determining the extent of application of the priorities, this study aimed to obtain an image of neglected areas in health research.

## Methodology

This was a retrospective real-world registry-based investigation that primarily describes and analyzes the state of clinical trials in relation to the most recently established national research priorities aiming to evaluate the effectiveness of research prioritization on guiding research in Iran.

### Introducing data and how to prepare them for analysis

To match clinical trials with research priorities, data related to the disease or conditions under study in the clinical trials were required. These data were extracted by observing the titles of each trial through the IRCT website’s search page located at http://www.irct.ir/search/ and subsequently recorded in an Excel file. In instances where identification of the condition under investigation through its title proved unfeasible, we referred to the study summary. In the event of persisting ambiguity, the ICD-10 code—a compulsory element for trial registration, available on the page of each respective trial within the IRCT website—was utilized. Concurrently, the start date of the study, patient recruitment, was also recorded.

Classification of the disease (conditions) under study was performed based on the country’s research priorities in the two areas of communicable and non-communicable diseases over five years running from 2009 to 2013 [6,23]. Some established research priorities were not suitable for conducting clinical trials (such as studies on the burden of diseases), which were removed from the list; also, putting "delinquency and behavioral disorders" and "High-risk behaviors of youth" in one category, a list of 10 priorities in communicable diseases and 10 priorities in non-communicable diseases was ultimately prepared (Table 1). For each of these conditions, a code from 1 to 20 was considered, and codes 21, 22, and 23 were also used for "other communicable diseases," "other non-communicable diseases," and "studies in other areas that do not include communicable and non-communicable diseases," respectively.

**Table 1:** The list of 10 country’s research priorities used in this study^a^.

Rank	Non-communicable diseases	Communicable diseases
1	Cardiovascular diseases	Antimicrobial resistances
2	Injuries caused by accidents and incidents	Zoonotic diseases
3	Drug abuse	Nosocomial infections
4	Tobacco use	AIDS
5	Diabetes	Hepatitis
6	Cancer	MDR-TB
7	Delinquency and behavioral disorders + High-risk behaviors of youth	Urinary tract infections
8	Infertility	Sexually transmitted diseases
9	Growth disorders in children	Waterborne communicable diseases
10	Osteoporosis	Cutaneous and visceral leishmaniosis

^**a**^ Reference: Owlia et al. Determining health research priorities in Iran. Journal of the School of Public Health and Institute of Public Health Research. 2011; 9(2): 9–20 [In Persian].

### Data analysis and providing results

The proportion of clinical trials consistent with the top 10 research priorities in each of the two areas of communicable and non-communicable diseases to all clinical trials conducted in these two areas was calculated, and the frequency of clinical trials conducted for each of these priorities was also calculated. Furthermore, the distribution of this frequency in the two time periods A: 2004–2008 and B: 2009–2013 the period for which prioritization was carried out was determined based on the start date of the study and compared with each other.

## Results

Table 2 shows the distribution of all clinical trials registered in IRCT according to the field of study and in two-time subgroups based on the trial start date (patient enrollment). In total, 5049 trials have been registered in IRCT until September 3, 2013, of which 722 were started in period A and 4324 in period B. 92.6% of the trials were conducted in the field of non-communicable diseases, 6.1% in the field of communicable diseases and 1.3% in other areas. The proportion of trials conducted in the field of communicable diseases to the total number of trials decreased from 8.9% in period A to 5.6% in period B. On the other hand, this proportion increased for non-communicable diseases from 89.8% in period A to 93.1% increased in period B.

**Table 2:** Distribution of clinical trials registered in IRCT from August 2008 to December 2013 according to the study area.

Condition	All trials (N = 5049) no. (%)	Start date(start recruitment)	
		2004–2008 (n = 722) no. (%)	2009–2013 (n = 4324) no. (%)
Communicable diseases	307 (6.1)	64 (8.9)	243 (5.6)
Non-communicable conditions^a^	4675 (92.6)	648 (89.8)	4024(93.1)
Other areas	67 (1.3)	10 (1.4)	57(1.3)

^a^ The predicted start date for the three studies was 2014

Table 3 shows the distribution of registered clinical trials in IRCT based on the top 10 research priorities of the country’s medical universities in the field of communicable diseases in two time periods. The most significant changes in the proportion of trials conducted in this field are a decrease in the proportion of trials on leishmaniasis to all trials in the field of communicable diseases from 18.8% in period A to 2.9% in period B; a decrease in this proportion for zoonotic diseases from 7.8% to 1.2%, and for hepatitis from 14% to 3.7%. On the other hand, this proportion has increased for some research priorities, including antimicrobial resistance from 3.1% to 13.2%, nosocomial infections from 9.4% to 23.0%, and HIV/AIDS from 3.1% to 6.6%. In period A, 62.5% of all trials in the field of communicable diseases were conducted on research priorities related to this field, which decreased to 56.4% in period B. There are no significant changes in other areas of communicable diseases.

**Table 3:** Distribution of clinical trials registered in IRCT from August 2008 to December 2013 according to research priorities in the area of communicable diseases.

Rank	Disease	Total trials in this area (n = 307) no. (%)	Start date(Start recruitment)	
			2004–2008 (n = 64) no. (%)	2009–2013 (n = 243) no. (%)
1	Cutaneous and visceral leishmaniasis	19 (6.2)	12 (18.8)	7 (2.9)
2	Antimicrobial resistances	34 (11.1)	2 (3.1)	32 (13.2)
3	Zoonotic diseases	8 (2.6)	5 (7.8)	3 (1.2)
4	Nosocomial infections	62 (20.2)	6 (9.4)	56 (23.0)
5	HIV & AIDS	18 (5.9)	2 (3.1)	16 (6.6)
6	Hepatitis	18 (5.9)	9 (14.0)	9 (3.7)
7	MDR-TB	1 (0.3)	0	1 (0.4)
8	Urinary tract infections	11 (3.6)	3 (4.7)	8 (3.3)
9	Sexually transmitted diseases	5 (1.6)	0	5 (2.1)
10	Waterborne communicable diseases	1 (0.3)	1 (1.6)	0
-	Total priorities of communicable diseases	177 (57.4)	40 (62.5)	137 (56.4)
-	Other communicable diseases	130 (42.6)	24 (37.5)	106 (43.6)

Among the top 10 research priorities in the field of communicable diseases, most studies conducted in period B were in the field of nosocomial infections with 56 studies (23.0%) followed by antimicrobial resistances with 32 studies (13.2%). On the contrary, the least of them were in zoonosis with 3 studies (1.2%), and MDR tuberculosis with 1 study (0.4%). No studies have been conducted in the field of waterborne diseases during this period (Table 3).

Likewise, in Table 4, the overall distribution of registered clinical trials in IRCT is presented based on the top 10 research priorities of the country’s medical universities in the field of non-communicable diseases and two time periods. The proportion of trials conducted on cardiovascular diseases to all trials in this field increased from 8.3% in period A to 11.2% in period B and for diabetes from 4.8% to 7.3%. In contrast, this proportion decreased for infertility from 6.2% to 3.4% and for growth disorders in children from 1.8% to 0.7%. There are no significant changes in other areas between the two time periods. In both periods, nearly 33% of clinical trials in the field of non-communicable diseases corresponded to the research priorities of this field.

**Table 4:** Distribution of clinical trials registered in IRCT from August 2008 to December 2013 according to research priorities in the area of non-communicable diseases.

Rank	Disease	Total trials in this area (n = 4675) no. (%)	Start date(Start recruitment)	
			2004–2008 (n = 648) no. (%)	2009–2013 (n = 4024) no. (%)
1	Cardiovascular diseases^a^	505 (10.8)	54 (8.3)	450 (11.2)
2	Injuries caused by accidents and incidents	188 (4.0)	26 (4.0)	162 (4.0)
3	Drug abuse	44 (0.9)	7 (1.1)	37 (0.9)
4	Prevention and control of tobacco use	8 (0.2)	3 (0.5)	5 (0.1)
5	Diabetes	326 (7.0)	31 (4.8)	295 (7.3)
6	Cancer	159 (3.4)	24 (3.7)	135 (3.4)
7	Delinquency and behavioral disorders + High-risk behaviors in youth	63 (1.4)	8 (1.2)	55 (1.4)
8	infertility	177 (3.8)	40 (6.2)	137 (3.4)
9	Growth disorders in children	39 (0.8)	12 (1.8)	27 (0.7)
10	Osteoporosis	25 (0.5)	6 (0.9)	19 (0.5)
-	Total priorities of non-communicable diseases	1534 (32.8)	211 (32.6)	1322 (32.8)
-	Other non-communicable diseases^b^	3141 (67.2)	437 (67.4)	2702 (67.2)

^a,b^ The start date of one study on cardiovascular disease and two studies on other non-communicable diseases was 2014

The highest number of trials among the top 10 research priorities in the field of non-communicable diseases in period B was conducted on cardiovascular diseases with 450 studies (11.2%), followed by diabetes with 295 studies (7.3%), and the lowest number of trials was related to prevention and control of tobacco use with 5 studies (0.1%) and osteoporosis with 19 studies (0.5%).

## Discussion

The findings of this study demonstrated that a small proportion of clinical trials conducted in Iran are in the field of communicable diseases. Only 6.1% of all registered trials in the IRCT were conducted in the field of communicable diseases, while 92.6% of them were in the field of non-communicable diseases, and this gap has been increasing in recent years.

Although there has been an increase in the proportion of clinical trials conducted in some research priorities such as antimicrobial resistance and nosocomial infections, many other priorities related to communicable diseases have not received sufficient attention. The proportion of studies conducted on leishmaniasis, hepatitis, and other zoonotic diseases has decreased significantly compared to the pre-prioritization period, despite their high priority ranking. In the case of MDR tuberculosis, which is seventh on the list of priorities, only one trial was conducted during the five-year prioritization period, and no study was conducted on waterborne diseases during this period.

While there is a need to conduct studies to control infectious diseases, including clinical trials to find new drugs, diagnostic methods, and vaccines, they are not resaving enough attention [1,2,8,11,24,25]. In the world, little research is done in the field of "forgotten diseases" such as AIDS, leishmaniasis, malaria, and tuberculosis, while these diseases cause many deaths in developing countries every year and impose a heavy burden on poor communities and populations [26,27] these inequalities exist not only among different countries but also among different communities in the same country, and few researches are conducted on health problems in poorer populations [2,8,10,28].

In terms of non-communicable diseases and conditions, this study showed a significant increase in the proportion of registered clinical trials in the IRCT related to cardiovascular disease and diabetes compared to the pre-prioritization period. However, the proportion of clinical trials conducted on infertility and growth disorders in children decreased compared to the pre-prioritization period, and there was little change in other areas.

While the prevention and control of tobacco use—the most important preventable risk factor for non-communicable diseases—ranks fourth among the ten research priorities in this field but has allocated the lowest proportion of clinical trials among research priorities in non-communicable diseases. So that during the five-year prioritization period, only five clinical trials (0.1% of all trials conducted in the field of non-communicable diseases) were conducted in this area. This implies the need for greater attention to prevention-related trials in the field of non-communicable diseases.

The findings of this study showed that, in general, trials conducted in the field of communicable diseases were more consistent with research priorities compared to trials conducted in the field of non-communicable diseases. However, the degree of compliance of the studies with the priorities in the prioritization period did not increase compared to the previous period, and it even decreased in the case of communicable diseases. Even though most of the priorities of 2009–2013, especially in the field of communicable diseases, were not introduced in the previous national prioritization study [29]. This indicates the need for more attention to national research priorities by researchers and financial supporters of studies, as well as an investigation into the reasons for the ineffectiveness of the prioritization.

The COVID-19 pandemic has demonstrated that when researchers clearly understand research priorities, they receive adequate attention. Our search using the keyword COVID-19 in the public title of the trials available in the IRCT database identified 739 clinical trials between February 19, 2020, the announced onset date of the COVID-19 epidemic in Iran, and August 26, 2023 [22]. Some of the most successful examples are the clinical trials, proving the safety and efficacy of two domestically produced COVID-19 vaccines in Iran that helped with vaccine equity [30,31]. Another example is Iran’s collaboration with international clinical trials, such as the Solidarity Clinical Trial for COVID-19 Treatments [32], that aimed to identify effective treatments for COVID-19. Some of the contributing factors to the more active involvement of the researchers in trying to solve the problems created by the COVID-19 pandemic were that policymakers and decision-makers adopted more effective, tangible, and practical methods to communicate their research requirements to the scientific community and researchers.

The prerequisite of any successftual prioritization is to have a plan to turn the priorities into actual research [7]. Otherwise, priorities may be overlooked and fail to yield the expected impact. In the case of comprehensive prioritizations that try to cover all research domains, it seems that the lack of support by specific prioritizations, which express an agenda for conducting studies with details, iould impede their effectiveness; Because it places researchers against overarching subjects rather than the specific questions rooted in genuine research needs within each respective field. In this regard, it is even possible to carry out prioritization within a particular academic discipline, according to the gaps of contemporary knowledge, to the extent of expressing the exact titles and type of studies required [9].

Our study demonstrated that the trial registration centers’ data can serve as a valuable tool for evaluating the effectiveness of research prioritization. The importance of this lies in the fact that due to a lack of appropriate information on the effectiveness of research prioritization, the efficacy of such studies is usually not evaluated, and their success rate remains uncertain [5]. The value of the data of these registration centers is more understood when considering that there may not currently be a comparable source of information on research activities as comprehensive, of high quality, and easily accessible as clinical trial registration centers.

Using data from registration centers to study clinical trials has advantages and limitations. Some advantages include having access to data from a large number of registered clinical trials, having information on trials that have not yet been published and may never be published, and the ability to study trials performed in specific areas related to health. However, these types of studies also come with operational challenges. One of the implementation problems is that registration databases sometimes do not provide information that is directly processable for a cumulative analysis. One reason is how the data is recorded; for example, trial protocol information may be recorded as free text in some variables. Analyzing this type of data requires various degrees of data munging, which can take a lot of time and energy [8].

In the case of our study, at first, IRCT managers provided researchers with a cumulative data file in CSV format containing the study conditions based on the ICD-10 code. However, upon examining this dataset, it was discovered that due to issues with the data structure, conducting cumulative analysis on it was not feasible. Specifically, the data associated with the ICD-10 code was only available in a separate file from the other data, without a common field, making it impossible to merge them. As a result, determining the study’s start date using this dataset wasn’t possible. Consequently, we abandoned this approach and extracted the required data from the IRCT search page, as outlined in the methodology.

Efforts have been made worldwide to facilitate research on the data of trial registration centers. To this end, ClinicalTrials.gov has made data directly amenable to analysis available to the public for download [33]. The preparation of this dataset has been made possible by an algorithm that provides uniformity and classification of recorded information regarding the study field of trials [20,34].

It may be argued that registered clinical trials are not a suitable tool for evaluating the effectiveness of research prioritizations because registration centers do not include information on other types of studies. However, it should be noted that clinical trials account for a large portion of health R&D globally [2,8], and in some studies, data from registration centers have been used as a criterion for research activities [8,10].

Our study has limitations that should be considered when interpreting its results: 1) As mentioned, data from clinical trial registration centers do not include other types of studies such as observational studies and systematic reviews; 2) Since most clinical trial registrations in IRCT occur after patient recruitment begins [21], many trials, especially those that have recently started, may not yet be registered and therefore were not included in this study; and 3) In this study, to make a comparison with the five-year period before the prioritization goal, the trials registered in IRCT whose start date was 2004–2008 were used, which included a total of 722 studies (Table 1). But since IRCT was established in 2008, many trials conducted during this period may not have been registered in IRCT.

## Conclusion

This study illuminates inadequacies in the effectiveness of Iran’s national research prioritization strategy, within communicable and non-communicable diseases domains. The spotlight on clinical trial research gaps underscores the need for policymakers, sponsors, and researchers to address deficiencies, particularly in communicable diseases. Policymakers are urged to explore alternative communication methods to ensure scientists’ awareness and alignment with national goals. The scientific communities play a key role in this aspect by conducting research to systematically identify efficient methods of communicating research priorities to scientists, to identify facilitators or barriers hindering the awareness or adherence of scientists to research priorities, and to guide research initiatives that are in line with these priorities.

To enhance the utilization of IRCT data, it is recommended to create the opportunity for conducting cumulative analyses. Additionally, registering various types of studies with the aim of establishing a comprehensive source for both completed and ongoing research is advisable.
